# Spatial and Temporal Variations in Stable Carbon (δ^13^C) and Nitrogen (δ^15^N) Isotopic Composition of Symbiotic Scleractinian Corals

**DOI:** 10.1371/journal.pone.0081247

**Published:** 2013-12-02

**Authors:** Sarah Nahon, Nicole B. Richoux, Joanna Kolasinski, Martin Desmalades, Christine Ferrier Pages, Gael Lecellier, Serge Planes, Véronique Berteaux Lecellier

**Affiliations:** 1 Laboratoire d’excellence « CORAIL », USR 3278 CNRS-EPHE, Centre de Recherches Insulaires et Observatoire de l’Environnement, Papetoai, Moorea, Polynésie Française; 2 Department of Zoology and Entomology, Rhodes University, Grahamstown, South Africa; 3 Department of Botany, IsoEnvironmental Laboratory, Rhodes University, Grahamstown, South Africa; 4 Laboratoire d'Ecologie Marine, Université de La Réunion, St Denis, La Réunion, France; 5 Centre Scientifique de Monaco, Principality of Monaco; 6 Université de Versailles, Saint Quentin en Yvelines, Versailles, France; University of Otago, New Zealand

## Abstract

Tropical scleractinian corals are considered autotrophic as they rely mainly on photosynthesis-derived nutrients transferred from their photosymbionts. Corals are also able to capture and ingest suspended particulate organic matter, so heterotrophy can be an important supplementary trophic pathway to optimize coral fitness. The aim of this *in situ* study was to elucidate the trophic status of 10 coral species under contrasted environmental conditions in a French Polynesian lagoon. Carbon (δ^13^C) and nitrogen (δ^15^N) isotopic compositions of coral host tissues and photosymbionts were determined at 3 different fringing reefs during wet and dry seasons. Our results highlighted spatial variability in stable isotopic compositions of both coral host tissues and photosymbionts. Samples from the site with higher level of suspended particulate matter were ^13^C-depleted and ^15^N-enriched relative to corals and photosymbionts from less turbid sites. However, differences in both δ^13^C and δ^15^N between coral host tissues and their photosymbionts (Δ^host-photosymbionts 13^C and Δ^host-photosymbionts 15^N) were small (0.27 ± 0.76‰ and 1.40 ± 0.90‰, respectively) and similar at all sites, thus indicating no general increases in the heterotrophic pathway. Depleted δ^13^C and enriched δ^15^N values of coral host tissues measured at the most turbid site were explained by changes in isotopic composition of the inorganic nutrients taken up by photosymbionts and also by changes in rate of isotopic fractionation with environmental conditions. Our results also highlighted a lack of significant temporal variations in δ^13^C and δ^15^N values of coral host and photosymbiont tissues and in Δ^host-photosymbionts 13^C and Δ^host-photosymbionts 15^N values. This temporal stability indicated that corals remained principally autotrophic even during the wet season when photosymbiont densities were lower and the concentrations of phytoplankton were higher. Increased coral heterotrophy with higher food availability thus appears to be species-specific.

## Introduction

Tropical scleractinian corals, which live in symbiosis with dinoflagellates of the genus S*ymbiodinium*, are extremely well adapted to their oligotrophic environment. The algal photosymbionts transfer a large fraction of the photosynthesis-derived carbon to their animal host and contribute significantly to its nutrition [1]. However, photosynthates translocated by photosymbionts are deficient in nitrogen, phosphorus and other nutrients [2], and the capture of suspended particulate organic matter (SPOM) including phytoplankton, zooplankton and detritus or/and the assimilation of dissolved inorganic and organic compounds is essential to optimize coral fitness [3]. Thus, scleractinian corals can be considered as opportunistic feeders that are able to use extremely diverse trophic pathways. These organisms assume several ecological roles simultaneously, spanning the levels of primary producer, herbivore, carnivore, detritivore and consumer of dissolved organic matter. In shallow waters, photosynthetic rates of photosymbionts are high [4] and scleractinian corals rely heavily on translocated photosynthates for their nutrient requirements [5–7]. At these depths, corals are principally autotrophic. In contrast, photosynthetic rates of photosymbionts in deep-water corals are low [4], much lower quantities of photosynthates are supposed to be produced and translocated, and hence corals are more heterotrophic [8]. However, corals do not shift from almost exclusive autotrophy in shallow water to heterotrophy in the deep reef [9]. Recent observations on numerous symbiotic coral species from temperate and tropical reefs support the idea that heterotrophy can be important at all depths, and it is well established that environmental factors such as light availability, seawater temperature, nutrient status and suspended particulate organic matter (SPOM) concentration all influence coral nutrition [3,8,10–13]. 

Stable carbon (δ^13^C) and nitrogen (δ^15^N) isotopic composition are useful measures for delineating carbon flow and tropic relationships in a large variety of continental and deep marine ecosystems [14,15]. Natural δ^13^C values identify the relative contributions of potential food sources, as consumers are slightly ^13^C-enriched relative to their diet [16]. The larger δ^15^N fractionation occurring at each trophic transfer (typically +2.3 ± 0.18‰ is assumed by McCutchan et al. [17]) allows us to infer important structural features of food webs such as the number of trophic levels [18] and the prevalence of omnivory [19]. Host tissues of autotrophic corals are generally slightly ^15^N-enriched and ^13^C-depleted compared to their photosymbiont as a result of isotopic fractionation associated with reciprocal exchanges of carbon and nitrogen between hosts and photosymbionts [8,20–22]. When the degree of heterotrophy by corals increases, the δ^13^C values of coral hosts and their photosymbionts become increasingly disparate and host signatures approach those of ^13^C-depleted heterotrophic sources (i.e. zooplankton prey and particulate organic matter with δ^13^C < -16‰) [8]. Details regarding the changes in δ^15^N values associated with higher degrees of heterotrophy remain elusive. However, δ^13^C values in coral host tissues relative to their photosymbionts can indicate the net translocation of photosynthates from the photosymbionts to the coral host under different environmental conditions. Both δ^13^C and δ^15^N values of scleractinian corals are influenced by additional factors such as the isotopic values of the dissolved inorganic carbon and nitrogen sources [23,24], nutrient concentrations [25], respiration rates [26], and light availability [8,13,21]. 

Numerous studies have focused on measurements of stable isotope composition of coral skeletal material, whereas few researchers have examined *in situ* natural variations of both δ^13^C and δ^15^N in coral host tissues and photosymbionts among coral reefs [23,27], and even fewer have made these measurements in different seasons on several coral species [22,28,29]. For example, Swart et al. [22,28] showed clear seasonal variations of δ^13^C in the coral *Montastraea faveolata* at a few reefs in Florida. An important challenge is to further refine our understanding of the effects of changing environmental factors on the trophic role of different coral species. Stable isotope ratios represent a suitable tool with which we can address this challenge, and as more data are produced we can improve on our ability to accurately interpret feeding relationships in complex symbiotic organisms. Furthermore, significant differences in both δ^13^C and δ^15^N values among coral species living in the same environment have been highlighted [8,21], and the sources of these differences require further attention. Interspecific variations have been attributed to differences in diffusion distance driving exchange rates between internal and seawater dissolved inorganic nutrient and/or differences in feeding rates [8,9,13,21,25,30]. 

The aims of this study were to investigate the spatial and temporal variations in δ^13^C and δ^15^N values of scleractinian coral host tissues and their photosymbionts from Moorea Lagoon (Society Island, French Polynesia). We hypothesized that (1) corals living in turbid fringing reefs with high levels of both suspended particulate inorganic (SPIM) and organic (SPOM) matter rely more on heterotrophic resources than corals living in reefs with clear conditions, and that (2) corals are more heterotrophic during the cloudy wet season when solar radiation reaching the sea surface is minimal and SPOM concentration is higher. Ten species of scleractinian corals (representing six genera) were sampled in three sites of Moorea Lagoon and during both wet and dry season to determine the interspecific variability in coral trophic status. 

## Materials and Methods

### Study sites and coral sampling

**Figure 1:**
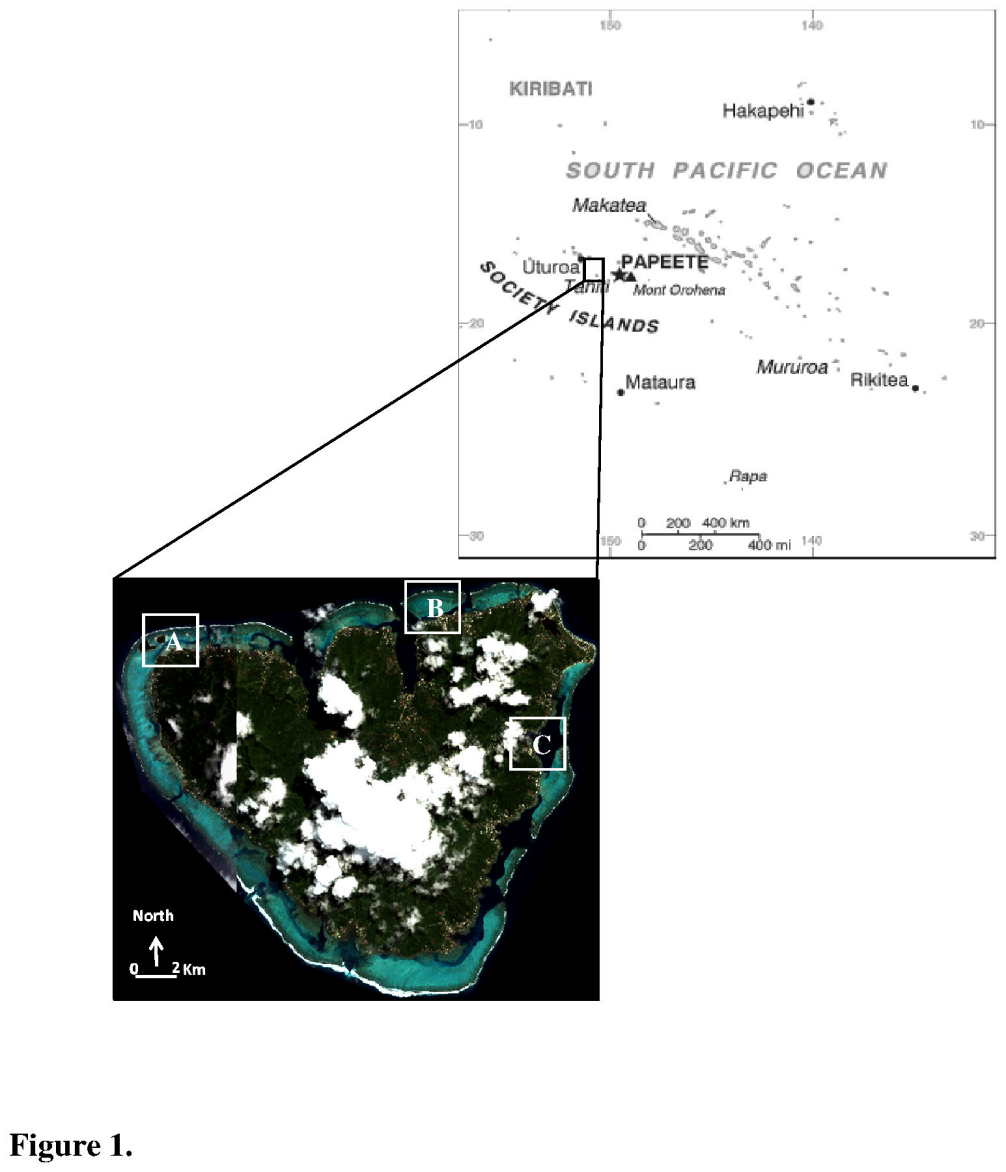
Map of the study sites. Localization of the reef of Tiahura (A), Maharepa (B) and Vaiare (C) in Moorea Island (Archipelago of Society, French Polynesia).

The study was conducted on three fringing reefs in Moorea Lagoon, French Polynesia (Figure **1**). The reef of Tiahura (17°29'24S, 149°53'57W) is well preserved, whereas the reefs of Maharepa (17°28'51S, 149°48'15W) and Vaiare (17°31'11S, 149°46'38W) are under growing anthropogenic pressures. Maharepa is the most urbanized city of the island and the reef is under the influence of two major rivers where wastewaters are released. Vaiare is the ferry area, a very turbid environment.

At least three colonies of the most abundant coral species (Table **1**) living between 0.5 and 1 m depth at Moorea Island were tagged *in situ* at each reef. Five fragments from each coral colony (5-10 cm^2^ ; fragments were collected to integrate the intra-colony variability [30]) were hand collected haphazardly over three days in March (wet season) and September (dry season) 2011. The same colonies were sampled during both seasons. Fragments were rinsed with 0.2 µm filtered seawater (FSW) and immediately frozen at -40°C until analysis. This field research was performed under annual research permits (unnumbered) issued by the French Polynesian Ministry of Research to the Centre de Recherches Insulaires et Observatoire de l'Environnement (CRIOBE). Approval was granted from our institutional animal ethics committee (Centre National de la Recherche Scientifique).

**Table 1:** List of coral species sampled at each site.

	Tiahura	Maharepa	Vaiare
Species	n		
*Porites rus*	5	5	5
*Napopora irregularis*	5	4	0
*Acropora cytherea*	3	0	3
*Acropora hyacinthus*	3	0	3
*Acropora pulchra*	3	5	3
*Pocillopora damicornis*	4	3	3
*Pocillopora meandrina*	4	0	3
*Pocillopora verrucosa*	3	4	4
*Pavona cactus*	4	3	4
*Montipora tuberculosa*	4	3	3

^n^ indicates the number of coral colonies sampled.

### Environmental parameters

Seawater parameters around the coral colonies were monitored at Tiahura, Maharepa and Vaiare during both sampling periods. Temperature, salinity and pH were recorded in triplicate using a YSI85 multi-parameter probe. Daily rainfall was measured at the Meteo France Station in Opunohu Bay (Moorea Island) and then cumulated for the 6 months wet season (November to April) and the 6 months dry season (May to October). Seawater samples (40 ml, 20 ml and 20 ml) were collected in triplicate to determine the concentrations of ammonium (NH_4_^+^), phosphate (PO_4_^-^) and the pools of nitrate (NO_3_^-^), nitrite (NO_2_^-^) and silicate (SiO_4_), respectively. NH_4_^+^ and PO_4_^-^ were analyzed immediately after sampling, whereas seawater samples intended for NO_3_^-^, NO_2_^-^ and SiO_4_ analyses were preserved with HgCl_2_^-^ and analyzed later by colorimetry using standard techniques [31] and a Technicon Auto-analyzer II. NH_4_^+^ concentrations were determined with a Turner Design TD-700 using the fluorometric and o-phthaldialdehyde method described in Holmes et al. [32]. PO_4_^-^concentrations were measured with a Cecil-CE 1011 spectrophotometer (cell length: 10 cm) using the molybdenum blue reaction [33]. 

Chlorophyll a (Chl *a*) was used as a proxy for phytoplankton biomass in the lagoon. Water samples (250 ml in triplicate) were vacuum-filtered onto Whatman GF/F filters (25 mm). Chlorophyll a was immediately extracted from each filter with 96% ethanol (5h, 4°C in dark) and analyzed using a Turner TD700 fluorometer calibrated with pure Chl *a* standard [34,35].

Water samples were collected with buckets (15 L) in triplicate to determine suspended particulate matter (SPM), suspended particulate inorganic matter (SPIM) and suspended particulate organic matter (SPOM) concentrations following Strickland and Pearson [31]. SPIM was a proxy for the levels of sedimentation and turbidity that impacted light penetration into seawater and coral physiology. Briefly, seawater was pre-filtered through a 200 μm mesh to remove large detritus and then filtered onto pre-combusted (4 h, 450°C) and pre-weighed Whatman GF/F filters (45 mm). Filters were rinsed with MilliQ water to remove salts and dried at 60°C for 24h before weighing to determine SPM concentrations. SPIM was determined by weight loss after ignition at 450°C for 5h. SPOM concentrations were calculated from the differences between SPM and SPIM. For stable isotope analysis of the SPOM, filters were prepared as described previously, acidified with 1N HCl, rinsed with MilliQ water and dried at 60°C.

### Coral preparations

Coral tissues were removed from the skeletons with an airbrush and approximately 50 ml of 0.2 µm FSW. The slurry was homogenized for 1 min in a blender Ultra-Turrax^®^ to release photosymbionts from the tissues. A subsample of each homogenate containing both coral host and photosymbionts cells was filtered on pre-combusted Whatman GF/F filters (45 mm) using low pressure. The volume of homogenates filtered varied from 1 to 10 ml according to the cell concentration of each sample. Filters were acidified briefly with 1N HCl to avoid any calcium carbonate contamination from the skeleton and to ensure that only organic carbon was analyzed. Then, samples were rinsed with MilliQ water and dried at 60°C until analysis for stable isotopes. Acidification effects on δ^13^C and δ^15^N values were assessed using a set of subsamples. As expected, acidification decreased δ^13^C values of samples containing carbonates. Similar to the findings of Heikoop et al. [36] and Muscatine et al. [37], our preliminary assessment showed that quickly rinsing the samples with weakly concentrated HCl (1N) did not significantly affect nitrogen isotope values, as the shift between acidified and untreated samples was within the error margin of the mass spectrometer. Hence, carbon and nitrogen isotopic compositions were measured on the same acidified sample. Two subsamples (250 µl) of homogenate were fixed with 4% formaldehyde and photosymbiont densities were estimated using a Malassez haemocytometer [38]. To determine Chl *a* concentrations in corals, 1 ml of each homogenate was filtered onto a Whatman GF/F filter (25 mm) and analyzed as described previously. Photosymbiont density and Chl *a* concentration were normalized to dry tissue biomass of corals (host tissues + algal photosymbionts) as recommended by Edmunds and Gates [39].

The remaining homogenate was divided into host and photosymbiont fractions by centrifugation (2000 x g, 5 min at 4°C) to pellet most of the photosymbionts [8]. This step was repeated 4 times to separate any remaining photosymbionts. The supernatant was checked under a microscope to confirm the purity of coral host tissues. For host tissue stable isotope measurements, the supernatant was prepared on pre-combusted Whatmann GF/F filters (45 mm) following the same treatment described previously for coral host and photosymbiont cells. Pellets of photosymbionts from the 4 centrifugations were pooled and diluted with 2 ml FSW. Photosymbionts were centrifuged (50 x g, 2 min at 4°C) and the 2 ml of supernatant containing coral host debris were discarded. Photosymbionts were cleaned following this method at least 6 times until almost no contamination by host cells was visible under a microscope. Clean photosymbionts were then prepared as per the coral host tissue and both coral host and photosymbiont samples for stable isotope analysis.

### Stable isotope analysis

Isotopic compositions (δ^13^C and δ^15^N) of all samples were analyzed using a Europa Scientific 20/20 isotope ratio mass spectrometer interfaced with an ANCA-SL elemental analyzer (continuous flow EA-IRMS). The ^13^C/^12^C and ^15^N/^14^N ratios are expressed in conventional delta notation in per mil (‰) relative to the levels of ^13^C in Vienna Pee Dee Belemnite and ^15^N in atmospheric air, according to the following equation:

$$\delta X=\left[\left(\frac{\text{Rsample}}{\text{Rstandart}}\right)-1\right]\times 1000$$

where X is ^13^C or ^15^N and R is the ratio of heavy to light isotope (^13^C/^12^C or ^15^N/^14^N). Repeated measurements of an internal standard exhibited a precision of ± 0.06‰ for δ^13^C and ± 0.12‰ for δ^15^N. In-house standards of beet sugar, ammonium sulfate and casein were calibrated against IAEA standards CH-6 and N-1.

### Statistical analysis

Non-parametric permutational multivariate analysis of variance (PermANOVA, statistic (F), degree of freedom (df)) were used to test for (1) spatial and temporal variations in environmental parameters and (2) species, spatial and temporal variations in photosymbiont density, Chl *a* concentration and stable isotope values of corals [40]. This method analyses the variance of multivariate data explained by a set of explanatory factors on the basis of Euclidean distances, so that effects linked to each factor or interactions between factors can be tested. Associated post-hoc tests (pair-wise comparisons) were completed to further explore significant interactions or main effects using Monte Carlo approximate *p*-values [40] when insufficient unique permutations existed for meaningful tests. Mann-Whitney tests (statistic (U), number of groups (N)) were realized to assess univariate temporal variations of environmental parameters and stable isotope values of corals at each site. Correlation analyses were performed on environmental parameters and coral data using Spearman's correlation coefficient. All statistical analyses were done using PRIMER 6 & PERMANOVA+ β17 and R (version 2.15.2).

## Results

### Environmental parameters and site characteristics

The dry season was characterized by an almost two times decrease in rainfall (755.5 mm and 449.8 mm for the 6 months of wet and dry seasons, respectively). At Moorea Island, seawater temperature was lower (28.5 ± 0.4°C and 26.9 ± 0.2°C, F = 9.41, df = 2, *p* ≤ 0.01) and salinity was higher (31.5 ± 0.0 and 35.7 ± 0.6, F = 28.5, df = 2, *p* ≤ 0.001) in the dry season relative to the wet season. Dissolved inorganic nitrogen (DIN) concentrations varied temporally according to the sites considered (F = 36.3, df = 2, *p* ≤ 0.01). Tiahura had lower nutrient concentrations than Maharepa and Vaiare (F = 49.8, post-hoc multiple comparison test, df = 2, *p* ≤ 0.001; Table **2**) and no significant temporal differences were observed (0.50 ± 0.05 and 0.51 ± 0.01 µM for wet and dry seasons, respectively, U = 0.4, N = 3, *p* ≤ 0.5). Conversely, during the dry season, DIN concentrations increased at Maharepa (0.59 ± 0.05 and 1.05 ± 0.07 µM for wet and dry, respectively, U = 3.8, N = 3, *p* ≤ 0.05), and decreased at Vaiare (0.87 ± 0.15 µM and 0.48 ± 0.02 µM for wet and dry, respectively, U = 4.0, N = 3, *p* ≤ 0.05). Phosphate concentrations were smaller and silicate concentrations were greater at all sites during the dry season (F = 7.8, df = 2, *p* ≤ 0.01).

**Table 2:** Nutrient concentrations in µM.

Site	Tiahura		Maharepa		Vaiare	
Season	Wet	Dry	Wet	Dry	Wet	Dry
NH_4_^+^	0.10 ± 0.01	0.11 ± 0.01	0.27 ± 0.05	0.24 ± 0.01	0.38 ± 0.07	0.21 ± 0.02
NO_2_^-^	0.02 ± 0.00	0.02 ± 0.00	0.02 ± 0.00	0.05 ± 0.00	0.04 ± 0.00	0.02 ± 0.00
NO_3_^-^	0.38 ± 0.12	0.37 ± 0.10	0.30 ± 0.01	0.76 ± 0.06	0.45 ± 0.09	0.25 ±0.02
PO_4_^-^	0.30 ± 0.03	0.23 ± 0.03	0.31 ± 0.03	0.18 ± 0.01	0.38 ± 0.06	0.30 ± 0.04
Si(OH)_4_	1.32 ± 0.14	2.36 ± 0.02	1.42 ± 0.09	2.81 ± 0.21	2.03 ± 0.05	2.88 ± 0.03

**Figure 2:**
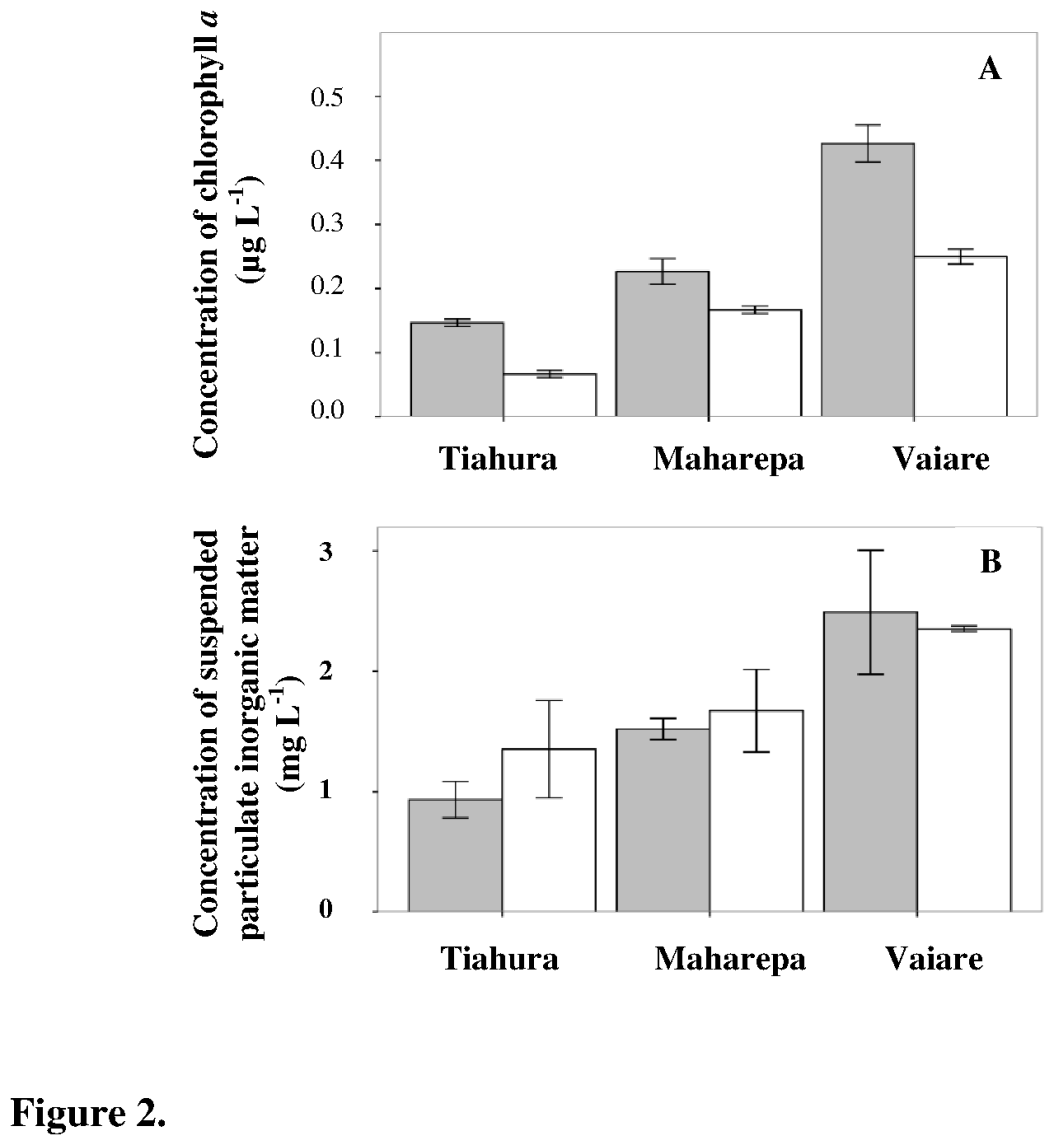
Environmental parameters: chlorophyll *a* and suspended particulate inorganic matter concentrations. Chlorophyll a (A) and suspended particulate inorganic matter (B) concentrations are expressed as mean ± standard deviation at Tiahura, Maharepa and Vaiare during both the wet (grey) and dry (white) seasons.

**Figure 3:**
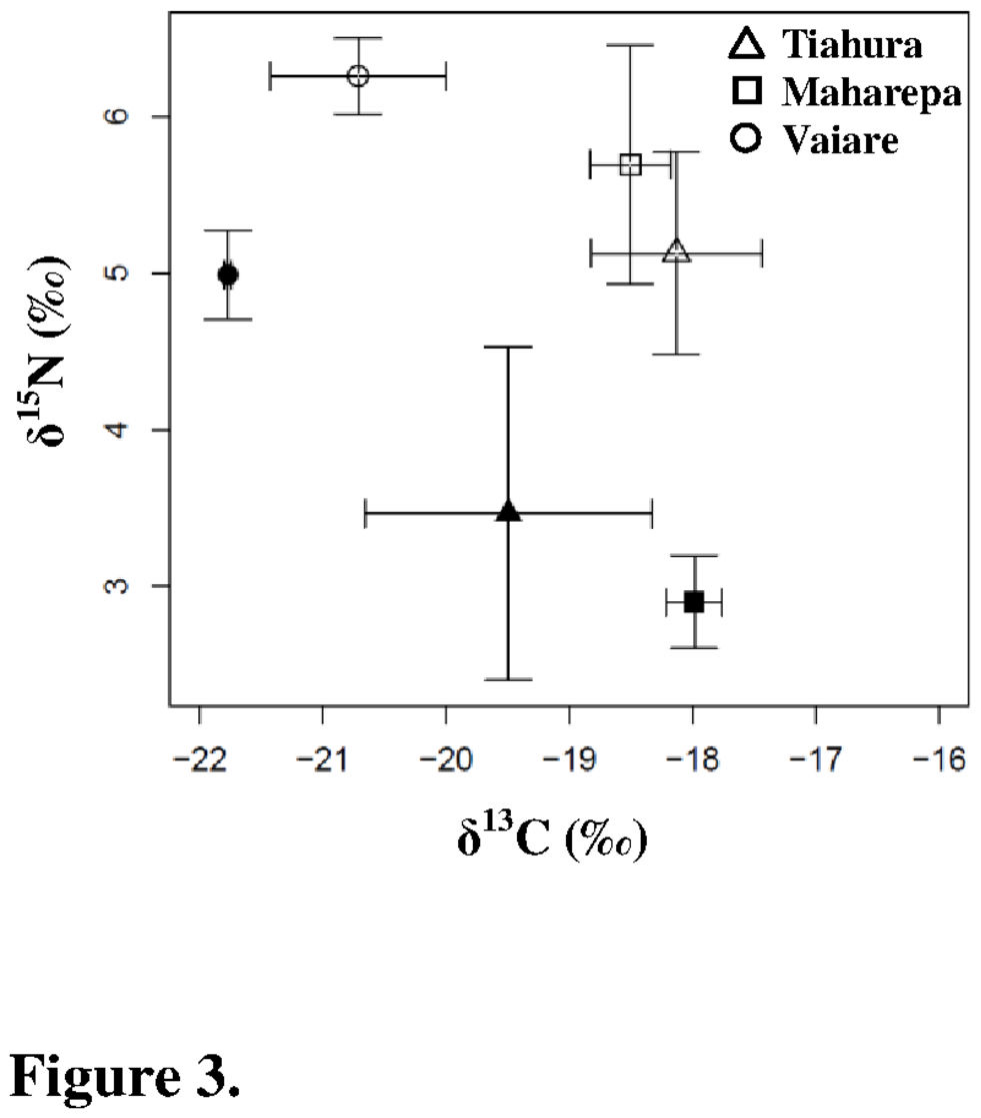
Environmental parameters: relationship between δ^13^C and δ^15^N of suspended particulate organic matter. δ^13^C and δ^15^N are expressed as mean ± standard deviation at all sites during both the wet (open symbols) and dry (black symbols) seasons.

**Figure 4:**
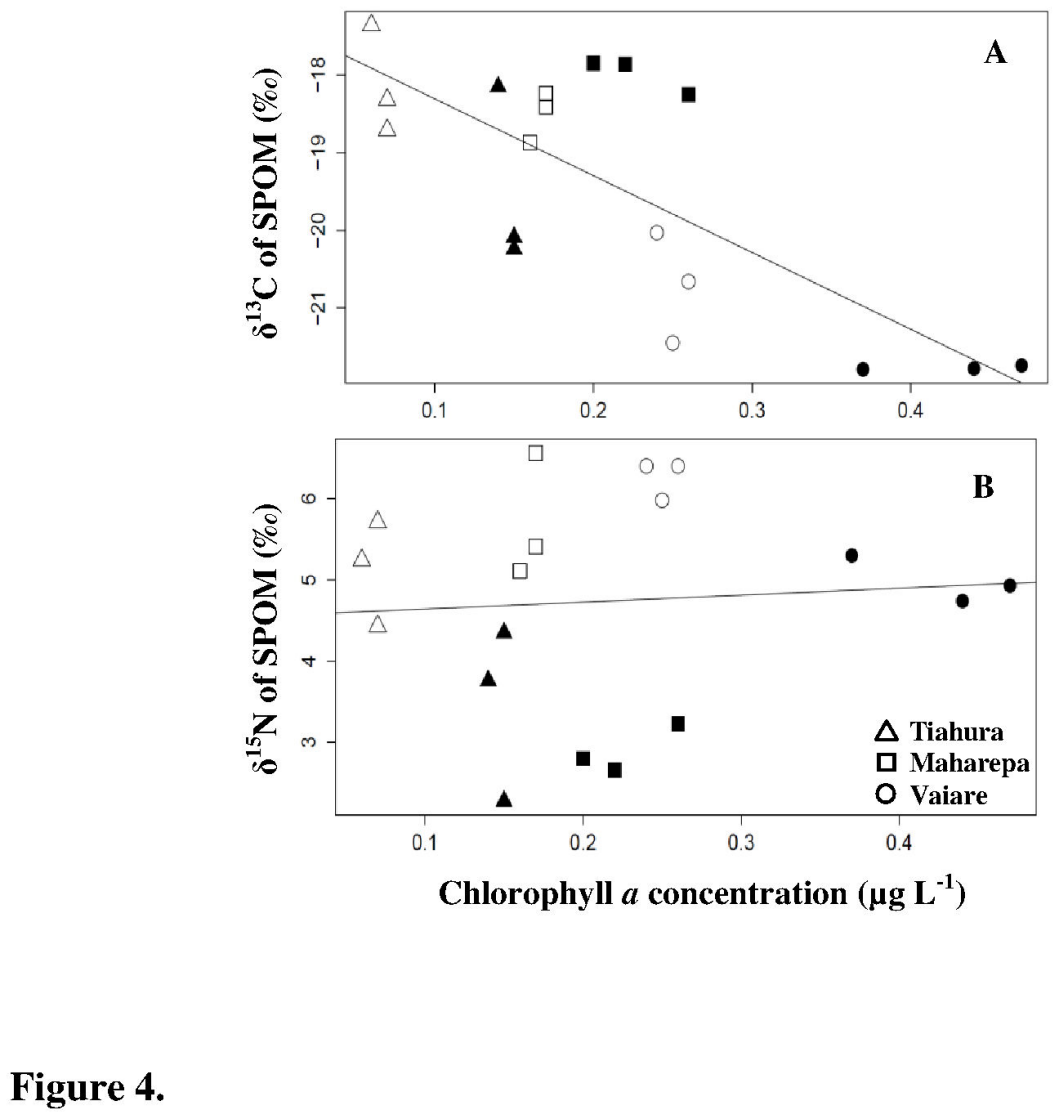
Environmental parameters: correlation between δ^13^C and δ^15^N of suspended particulate organic matter and chlorophyll *a* concentrations. Results are presented for all sites during both the wet (open symbols) and dry (black symbols) seasons, graphics A and B respectively. (A) δ^13^C = -17.22 - 9.88*Chl*a* (r = -0.59,N = 18, *p* ≤ 0.01), (B) non significant correlation (r = 0.13, N = 18, *p* = 0.6).

Chl *a* concentrations were higher at Vaiare compared with Maharepa and Tiahura (F = 128.7, df = 2, post-hoc multiple comparison test, *p* ≤ 0.001; Figure **2A**). During the dry season, Chl *a* concentrations were significantly lower at all sites (F = 9.3, df = 2, *p* ≤ 0.01). SPIM, a proxy for the levels of sedimentation and seawater turbidity, was higher at Vaiare than at Maharepa and Tiahura (F = 26.1, df = 2, Post-hoc multiple comparison test, *p* ≤ 0.01) during both collections, while no temporal variations were observed (F = 0.98, df = 1, *p* = 0.33; Figure **2B**). SPOM from Vaiare was more ^13^C-depleted and ^15^N-enriched than SPOM from Tiahura and Maharepa (F = 36.3 and F = 3.95 respectively, df = 2, post-hoc multiple comparison test, *p* ≤ 0.001; Figure **3**). No temporal effect was observed for δ^13^C values (F = 4.3, df = 2, *p* = 0.06), whereas some ^15^N-enrichment was apparent in SPOM during the dry season at all sites (F = 41.5, df = 2, *p* ≤ 0.001). δ^13^C (but not δ^15^N) values of SPOM were significantly correlated with Chl *a* concentrations (r = -0.59, *p* ≤ 0.01 and r = 0.13, *p* = 0.6, respectively; Figure **4A-B**).

### Variations of photosymbiont density and chlorophyll *a* concentrations in coral tissues

A strong positive correlation was noted between the density of photosymbionts and Chl *a* concentrations in coral tissues (Chl *a* = 3.5 x 10^-3^ photosymbiont density, r = 0.84, N = 192, *p* ≤ 0.001, graph not shown). Densities of photosymbionts varied from 0.44 ± 0.09 x 10^5^ cells mgDW^-1^ in *Montipora tuberculosa* at Tiahura during the wet season to 3.22 ± 0.68 x 10^5^ cells mgDW^-1^ in *Pocillopora damicornis* at Maharepa during the dry season (Table **3**). Considering all sites and both seasons, *Acropora pulchra* and *P. damicornis* had significantly more photosymbionts in their tissues than *Porites rus*, *Napopora irregularis*, *Acropora cytherea*, *Pocillopora meandrina*, *Pocillopora verrucosa* and *M. tuberculosa* (post-hoc multiple comparison test, *p* ≤ 0.001; Table **3** and Table **4**).

**Table 3:** Photosymbiont density (10^5^ cells mgDW^-1^) and chlorophyll *a* concentration (µg mgDW^-1^) in coral tissues (mean ± standard deviation) for all species at Tiahura, Maharepa and Vaiare during both wet and dry seasons.

**Site**	**Tiahura**				**Maharepa**				**Vaiare**				**Mean**	
**Season**	**Wet**		**Dry**		**Wet**		**Dry**		**Wet**		**Dry**			
**Species**	**Density**	**Chl *a***	**Density**	**Chl *a***	**Density**	**Chl *a***	**Density**	**Chl *a***	**Density**	**Chl *a***	**Density**	**Chl *a***	**Density**	**Chl *a***
***P. rus***	0.68 ± 0.17	573 ± 147	1.07 ± 0.13	473 ± 98	1.10 ± 0.35	790 ± 207	1.41 ± 0.25	874 ± 110	0.74 ± 0.18	362 ± 87	1.18 ± 0.20	547 ± 67	1.03 ± 0.33	597 ± 218
***N. irregularis***	1.05 ± 0.30	525 ± 147	0.85 ± 0.20	323 ± 82	1.19 ± 0.32	874 ± 236	1.59 ± 0.31	528 ± 142	n.d.	n.d.	n.d.	n.d.	1.14 ± 0.37	547 ± 245
***A. cytherea***	0.95 ± 0.20	324 ± 171	1.50 ± 0.15	531 ± 45	n.d.	n.d.	n.d.	n.d.	0.78 ± 0.26	319 ± 93	0.71 ± 0.11	269 ± 57	0.99 ± 0.36	361 ± 138
***A. hyacenthus***	0.91 ± 0.18	435 ± 104	1.27 ± 0.25	517 ± 71	n.d.	n.d.	n.d.	n.d.	0.96 ± 0.18	388 ± 15	1.23 ± 0.21	476 ± 72	1.09 ± 0.24	454 ± 101
***A. pulchra***	2.04 ± 0.32	778 ± 186	1.70 ± 0.31	530 ± 95	1.75 ± 0.11	815 ± 96	2.28 ± 0.44	649 ± 194	1.76 ± 0.45	474 ± 94	2.97 ± 0.28	643 ± 63	2.07 ± 0.52	663 ± 171
***P. damicornis***	1.89 ± 0.48	641 ± 143	1.60 ± 0.37	620 ± 102	2.66 ± 0.38	879 ± 131	3.22± 0.68	376 ± 31	1.02 ± 0.12	298 ± 60	1.61 ± 0.11	554 ± 105	2.03 ± 0.90	568 ± 207
***P. meandrina***	0.66 ± 0.11	328 ± 78	0.99 ± 0.11	391 ± 136	n.d.	n.d.	n.d.	n.d.	0.29 ± 0.03	209 ± 32	0.77 ± 0.21	507 ± 79	0.70 ± 0.03	597 ± 218
***P. verruculosa***	0.99 ± 0.22	459 ± 66	1.31 ± 0.41	593 ± 186	1.09 ± 0.35	510 ± 98	1.09 ± 0.29	282 ± 91	0.88 ± 0.16	299 ± 77	1.70 ± 0.35	592 ± 170	1.18 ± 0.39	449 ± 168
***P. cactus***	1.08 ± 0.16	452 ± 68	1.55 ± 0.16	479 ± 39	1.33 ± 0.41	365 ± 123	1.83 ± 0.58	703 ± 162	1.39 ± 0.57	474 ± 160	1.15 ± 0.20	324 ± 42	1.37 ± 0.41	460 ± 149
***M. tuberculosa***	0.44 ± 0.09	263 ± 64	0.81 ± 0.11	422 ± 81	1.05 ± 0.21	686 ± 213	1.02 ± 0.26	842 ± 234	1.06 ± 0.38	527 ± 173	1.34 ± 0.30	371 ± 34	0.92 ± 0.03	501 ± 235
**Mean**	1.04 ± 0.53	479 ± 182	1.23 ± 0.37	473 ± 126	1.42 ± 0.58	716 ± 228	1.79 ± 0.85	615 ± 249	0.98 ± 0.47	372 ± 135	1.39 ± 0.64	479 ± 142	1.29 ± 0.63	513 ± 206

n.d. means not detected when coral species was absent.

**Table 4:** Significance of species, spatial and temporal effects on photosymbiont density and chlorophyll *a* concentration in coral tissues and isotopic composition of corals tested with PermANOVA.

	*Photosymbiont density*		*Chl a concentration*		*δ^13^C of photosymbiont and polyp*		*δ* ^*15*^ *N of photosymbiont and polyp*		*δ^13^C of photosymbiont*		*δ^15^N of photosymbiont*		*δ^13^C of polyp*		*δ^15^N of polyp*		Δ^host-photosymbionts 13^C		Δ^host-photosymbionts 15^N	
	F	*p*	F	*p*	F	*p*	F	*p*	F	*p*	F	*p*	F	*p*	F	*p*	F	*p*	F	*p*
	(df)		(df)		(df)		(df)		(df)		(df)		(df)		(df)		(df)		(df)	
Species	45.5	***	6.9	***	73.5	***	17.8	***	102.7	***	53.2	***	95.2	***	27.4	***	35.3	***	82.7	***
	(2)		(9)		(9)		(9)		(9)		(9)		(9)		(9)		(9)		(9)	
Site	21.4	***	38.7	***	124.6	***	340.2	***	158.7	***	382.8	***	185.5	***	343.7	***	15.9	***	2.2	n.s.
	(2)		(2)		(2)		(2)		(2)		(2)		(2)		(2)		(2)		(2)	
Season	52.2	***	0.0	n.s.	0.6	n.s.	12.8	**	20.5	***	10.2	**	23.9	***	1.9	n.s.	465.3	***	1.8	n.s.
	(1)		(1)		(1)		(1)		(1)		(1)		(1)		(1)		(1)		(1)	
Site*Season	2.8	n.s.	10.1	***	6.7	**	0.0	n.s.	21.4	***	3.9	*	18.0	***	11.4	***	61.6	***	2.2	n.s.
	(2)		(2)		(2)		(2)		(2)		(2)		(2)		(2)		(2)		(2)	
Site*Species	9.8	***	4.9	***	10.4	***	1.9	*	12.9	***	3.7	***	8.3	***	3.7	***	27.9	***	8.3	***
	(14)		(14)		(14)		(14)		(14)		(14)		(14)		(14)		(14)		(14)	
Season*Species	0.6	n.s.	3.0	**	2.4	***	2.4	*	11.7	***	4.5	***	6.6	***	2.09	*	29.7	***	3.7	***
	(9)		(9)		(9)		(9)		(9)		(9)		(9)		(9)		(9)		(9)	
Season*Site*Species	4.0	***	6.0	***	2.5	**	1.0	n.s.	9.4	***	2.7	***	11.1	***	0.9	n.s.	22.6	***	12.1	***
	(14)		(14)		(14)		(14)		(14)		(14)		(14)		(14)		(14)		(14)	
Residuals	140		140		140		140		140		140		140		140		140		140	

* means *p* ≤ 0.5, ** *p* ≤ 0.01, *** *p* ≤ 0.001 and n.s. not significant.

When differences were significant, effects were further explored with associated post-hoc tests.

Densities of photosymbionts and Chl *a* concentrations were both significantly greater at Maharepa than at Tiahura and Vaiare (post-hoc multiple comparison test, *p* ≤ 0.001; Table **3** and Table **4**). A significant increase in photosymbiont density was observed during the dry season, but no temporal differences were apparent in Chl *a* concentrations of photosymbionts (Table **4**). For each coral species, spatial and temporal variations (and the interaction between factors site and season) were tested independently showing that differences among sites and between seasons were species-specific (Table **5**).

**Table 5:** Significance of spatial and temporal variations of photosymbiont density and chlorophyll *a* concentration in coral tissues tested with PermANOVA for each species.

Species	*P. rus*		*N. irregularis*		*A. cytherea*		*A. hyacinthus*		*A. pulchra*		*P. damicornis*		*P. meandrina*		*P. verrucosa*		*P. cactus*		*M. tuberculosa*	
	F	*p*	F	*p*	F	*p*	F	*p*	F	*p*	F	*p*	F	*p*	F	*p*	F	*p*	F	*p*
	(df)		(df)		(df)		(df)		(df)		(df)		(df)		(df)		(df)		(df)	
Photosymbiont density																				
Site	7.5	***	10.9	**	19.2	**	0.0	n.s.	3.6	n.s.	25.4	***	18.7	***	0.9	n.s.	1.3	n.s.	11.5	**
	(2)		(1)		(1)		(1)		(2)		(2)		(1)		(2)		(2)		(2)	
Season	20.6	***	0.2	n.s.	4.6	n.s.	6.7	*	11.2	**	2.6	n.s.	34.0	***	8.6	*	1.9	n.s.	4.6	n.s.
	(1)		(1)		(1)		(1)		(1)		(1)		(1)		(1)		(1)		(1)	
Site*Season	0.2	n.s.	5.2	*	7.8	n.s.	0.1	n.s.	8.2	**	3.2	n.s.	1.2	n.s.	3.6	n.s.	2.4	n.s.	1.3	n.s.
	(2)		(1)		(1)		(1)		(2)		(2)		(1)		(2)		(2)		(2)	
Residuals	24		14		8		8		16		14		10		16		16		14	
Chlorophyll a concentration																				
Site	3.6	n.s.	13.9	***	4.9	n.s.	0.5	n.s.	3.03	n.s.	7.7	**	0.0	n.s.	2.0	n.s.	2.8	n.s.	14.9	**
	(2)		(1)		(1)		(1)		(2)		(2)		(1)		(2)		(2)		(2)	
Season	11.2	**	13.0	**	1.7	n.s.	2.0	n.s.	2.7	n.s.	2.9	n.s.	10.6	*	1.3	n.s.	1.1	n.s.	0.9	n.s.
	(1)		(1)		(1)		(1)		(1)		(1)		(1)		(1)		(1)		(1)	
Site*Season	8.2	**	0.9	n.s.	4.5	n.s.	0.0	n.s.	4.0	*	19.6	***	5.3	n.s.	9.3	**	9.0	***	2.4	n.s.
	(2)		(1)		(1)		(1)		(2)		(2)		(1)		(2)		(2)		(2)	
Residuals	24		14		8		8		16		14		10		16		16		14	

* means *p* ≤ 0.5, ** *p* ≤ 0.01, *** *p* ≤ 0.001 and n.s. not significant.

### Variations in δ^13^C and δ^15^N values of scleractinian corals in relation to their associated photosymbionts

#### δ^13^C and δ^15^N values of coral hosts

**Figure 5:**
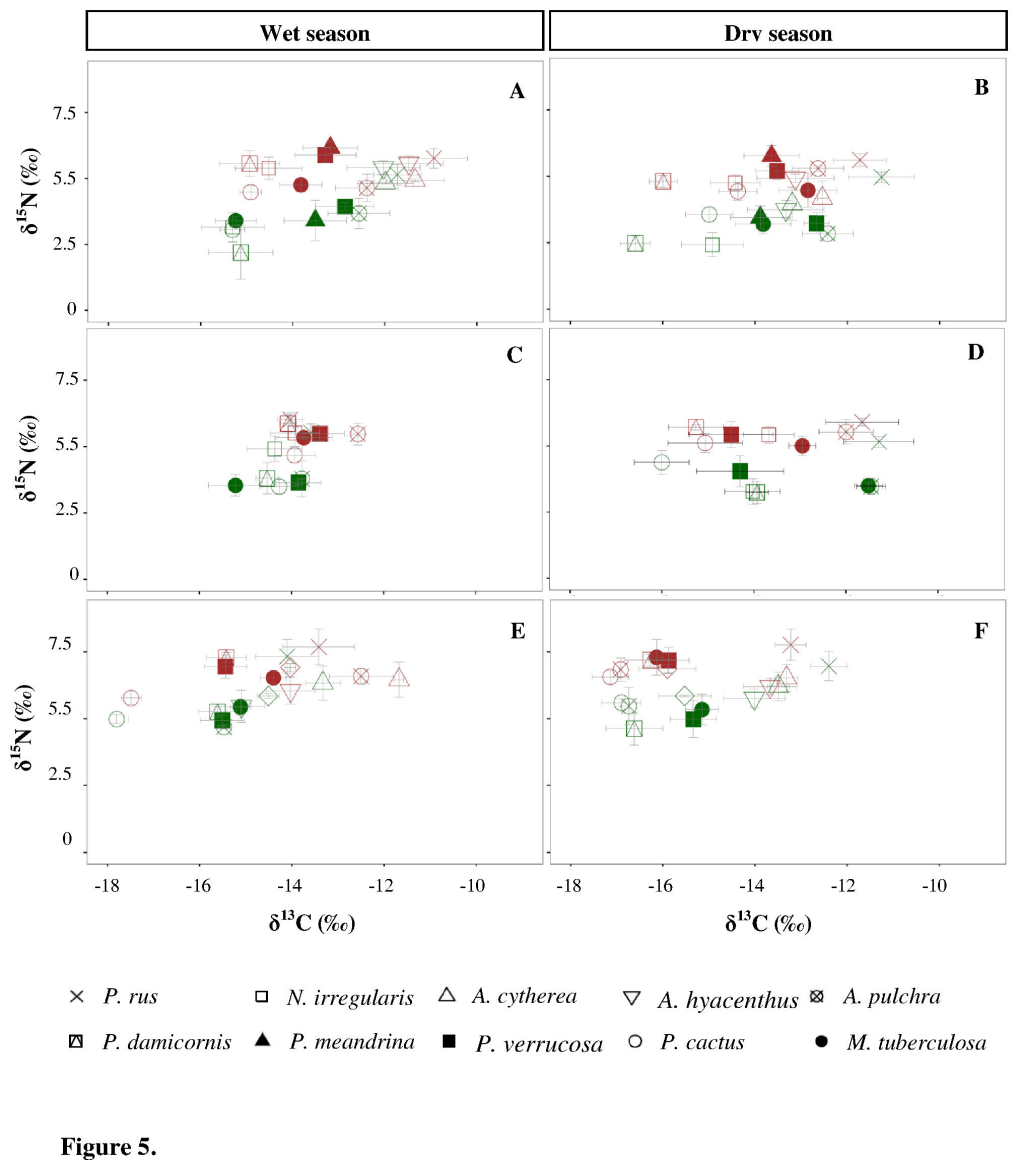
Variations in δ^13^C and δ^15^N isotope signatures of scleractinian corals. δ^13^C *versus* δ^15^N (mean ± standard deviation) for coral host tissues in brown and photosymbionts in green at Tiahura (A-B), Maharepa (C-D) and Vaiare (E-F) during both wet and dry seasons. Each coral species is represented by a symbol.

Isotopic composition of coral host tissues ranged from -17.5 ± 0.2‰ (*P. cactus* at Vaiare during the wet season) to -10.9 ± 0.7‰ (*P. rus* at Tiahura during the wet season) for δ^13^C, and from 4.2 ± 0.7‰ (*A. cytherea* at Tiahura during the dry season) to 7.8 ± 0.6‰ (*P. rus* at Vaiare during the wet season) for δ^15^N (Table **6** and Figure **5**). δ^13^C and δ^15^N values of coral host tissues varied significantly among species (Table **4**). Considering all sites and both seasons, δ^13^C values of coral host tissues from *P. cactus* and *P. damicornis* were more depleted than those of *A. cytherea*, *A. hyacinthus*, *A. pulchra* and *P. rus* (post-hoc multiple comparison test, *p* ≤ 0.05). *Porites rus* and *P. meandrina* were more ^15^N-enriched than *N. irregularis*, *P. cactus* and *M. tuberculosa* (post-hoc multiple comparison test, *p* ≤ 0.05).

**Table 6:** δ^13^C and δ^15^N values of corals (mean ± standard deviation) for all species at Tiahura, Maharepa and Vaiare during both wet and dry seasons.

**Site**	Tiahura				Maharepa								Vaiare								Mean	
**Season**	Wet		Dry		Wet				Dry				Wet				Dry					
**Species**	δ^13^C (‰)	δ^15^N (‰)	δ^13^C (‰)	δ^15^N (‰)	δ^13^C (‰)			δ^15^N (‰)	δ^13^C (‰)		δ^15^N (‰)		δ^13^C (‰)		δ^15^N (‰)			δ^13^C (‰)		δ^15^N (‰)	δ^13^C (‰)	δ^15^N (‰)
***P. rus***																						
Polyp and photosymbiont	-11.5 ± 0.3	5.3 ± 0.2	-11.6 ± 0.5	5.5 ± 0.3	-13.4 ± 0.3			5.6 ± 0.4	-11.4 ± 1.0		5.3 ± 0.3		- 12.3 ± 0.7		7.2 ± 0.4			-12.6 ± 1.0		6.9 ± 0.4	-12.1 ± 0.9	5.9 ± 0.8
Photosymbiont	-11.7 ± 0.5	5.1 ± 0.4	-11.3 ± 0.7	5.0 ± 0.2	-13.6 ± 0.2			5.5 ± 0.3	-11.3 ± 0.8		5.2 ± 0.2		-14.1 ± 0.7		7.3 ± 0.6			-12.4 ± 0.4		7.0 ± 0.5	-12.4 ± 1.2	5.8 ± 1.0
Polyp	-10.9 ± 0.7	5.8 ± 0.4	-11.7 ± 0.6	5.6 ± 0.2	-14.0 ± 0.3			6.0 ± 0.2	-11.6 ± 0.8		5.9 ± 0.1		-13.4 ± 0.8		7.7 ± 0.7			-13.2 ± 0.3		7.8 ± 0.6	-12.5 ± 1.3	6.5 ± 1.0
Δ^host-photosymbionts^	0.80 ± 0.89	0.62 ± 0.15	-0.47 ± 0.20	0.64 ± 0.17	-0.45 ± 0.11			0.49 ± 0.09	-0.37 ± 0.12		0.74 ± 0.14		0.69 ± 0.17		0.35 ± 0.10			-0.83 ± 0.11		0.80 ± 0.15	-0.10 ± 0.72	0.61 ± 0.20
***N. irregularis***																						
Polyp and photosymbiont	-15.3 ± 0.3	4.7 ± 0.2	-14.8 ± 0.7	4.2 ± 0.3	-13.9 ± 0.8			4.5 ± 0.5	-13.6 ± 0.6		4.3 ± 0.4		n.d.		n.d.			n.d.		n.d.	-14.5 ± 0.9	4.4 ± 0.4
Photosymbiont	-15.3 ± 0.7	3.1 ± 0.6	-14.9 ± 0.7	2.4 ± 0.4	-14.4 ± 0.6			4.9 ± 0.5	-14.0 ± 0.6		3.3 ± 0.5		n.d.		n.d.			n.d.		n.d.	-14.7 ± 0.8	3.4 ± 1.0
Polyp	-14.5 ± 0.7	5.4 ± 0.4	-14.4 ± 0.5	4.8 ± 0.3	-13.9 ± 0.5			5.5 ± 0.5	-13.7 ± 0.6		5.4 ± 0.3		n.d.		n.d.			n.d.		n.d.	-14.1 ± 0.6	5.2 ± 0.5
Δ^host-photosymbionts^	0.78 ± 0.25	2.24 ± 0.69	0.49 ± 0.14	2.33 ± 0.43	0.44 ± 0.07			0.59 ± 0.04	0.35 ± 0.09		2.14 ± 0.56		n.d.		n.d.			n.d.		n.d.	0.53 ± 0.22	1.87 ± 0.91
***A. cytherea***																						
Polyp and photosymbiont	-11.3 ± 0.5	4.8 ± 0.2	-12.5 ± 1.1	4 .4 ± 0.3	n.d.			n.d.	n.d.		n.d.		-12.2 ± 0.5		7.0 ± 0.7			-13.2 ± 0.2		6.4 ± 0.8	-12.3 ± 0.9	5.6 ± 1.2
Photosymbiont	-12.0 ± 0.6	4.8 ± 0.0	-13.2 ± 0.1	4.0 ± 0.6	n.d.			n.d.	n.d.		n.d.		-13.3 ± 0.4		6.3 ± 0.6			-13.5 ± 0.2		6.2 ± 0.5	-12.9 ±0.7	5.3 ± 1.1
Polyp	-11.3 ± 0.6	4.9 ± 0.0	-12.5 ± 0.1	4.2 ± 0.7	n.d.			n.d.	n.d.		n.d.		-11.7 ± 0.1		6.4 ± 0.6			-13.3 ± 0.2		6.5 ± 0.5	-12.2 ± 0.9	5.2 ± 1.1
Δ^host-photosymbionts^	0.64 ± 0.04	0.10 ± 0.03	0.66 ± 0.15	0.21 ± 0.05	n.d.			n.d.	n.d.		n.d.		1.65 ± 0.038		0.12 ± 0.00			0.18 ±0.02		0.32 ± 0.02	0.78 ± 0.59	0.19 ± 0.09
***A. hyacinthus***																						
Polyp and photosymbiont	-11.7 ± 0.7	5.7 ± 0.4	-13.1 ± 0.9	4.7 ± 0.4	n.d.			n.d.	n.d.		n.d.		-14.7 ±0.8		6.5 ± 0.7			-15.1 ± 1.0		5.7 ± 0.9	-13.6 ± 1.6	5.7 ± 0.9
Photosymbiont	-12 .0 ± 0.8	5.4 ± 0.2	-13.3 ± 0.8	3.7 ± 0.3	n.d.			n.d.	n.d.		n.d.		-15.1 ± 0.5		5.5 ± 0.6			-14.0 ± 0.7		5.8 ± 0.3	-13.6 ± 1.3	5.1 ± 0.9
Polyp	-11.5 ± 0.7	5.6 ± 0.3	-13.1 ± 0.8	4.9 ± 0.1	n.d.			n.d.	n.d.		n.d.		-14.0 ± 0.5		6.0 ± 0.5			-13.7 ± 0.6		6.2 ± 0.3	-13.1 ± 1.2	5.6 ± 0.6
Δ^host-photosymbionts^	0.55 ±0.05	0.16 ± 0.05	0.20 ± 0.02	1.18 ± 0.20	n.d.			n.d.	n.d.		n.d.		1.06 ± 0.30		0.59 ± 0.15			0.34 ± 0.08		0.44 ± 0.08	0.54 ± 0.37	0.59 ± 0.40
***A. pulchra***																						
Polyp and photosymbiont	-12.0 ± 0.8	5.2 ± 0.0	-11.9 ± 0.7	4.9 ± 0.2	-12.1 ± 0.4			5.0 ± 0.3	-11.1 ± 1.0		4.6 ± 0.3		-15.2 ± 0.4		6.2 ± 0.1			-16.4 ± 0.1		6.3 ± 0.3	-12.9 ± 2.0	5.3 ± 0.7
Photosymbiont	-12.6 ± 0.7	3.7 ± 0.6	-12.4 ± 0.5	2.8 ± 0.3	-13.8 ± 0.1			3.8 ± 0.7	-11.4 ± 0.3		3.5 ± 0.3		-15.5 ± 0.1		4.7 ± 0.1			-16.7 ± 0.0		5.5 ± 0.7	-13.5 ± 1.8	3.9 ± 0.9
Polyp	-12.4 ± 0.7	4.6 ± 0.5	-12.6 ± 0.6	5.3 ± 0.1	-12.6 ± 0.1			5.5 ± 0.4	-12.0 ± 0.6		5.5 ± 0.5		-12.5 ± 0.7		6.6 ± 0.3			-16.9 ± 0.0		6.8 ± 0.4	-13.0 ±1.7	5.7 ± 0.8
Δ^host-photosymbionts^	0.17 ± 0.02	0.96 ± 0.11	-0.21 ± 0.03	2.46 ± 0.24	1.21 ± 0.09			1.68 ± 0.33	-0.54 ± 0.36		2.06 ± 0.55		2.98 ± 0.58		3.90 ± 0.28			-0.18 ± 0.02		1.37 ± 0.38	0.53 ± 1.21	2.03 ± 0.94
***P. damicornis***																						
Polyp and photosymbiont	-14.9 ± 0.7	4.6 ± 0.5	-15.9 ± 0.3	4.3 ± 0.3	-14.1 ± 0.2			4.9 ± 0.2	-14.6 ± 0.4		4.9 ±0.2		-15.8 ± 0.8		6.7 ± 0.4			-16.5 ± 0.2		6.3 ± 0.4	-15.3 ± 0.9	5.2 ± 0.9
Photosymbiont	-15.1 ± 0.7	2.2 ± 1.0	-16.6 ± 0.3	2.5 ± 0.3	-14.5 ± 0.2			5.7 ± 0.2	-13.9 ± 0.2		3.2 ± 0.4		-15.6 ± 0.4		5.3 ± 0.2			-16.6 ± 0.6		4.6 ± 0.6	-15.5 ± 1.1	3.5 ± 1.2
Polyp	-14.9 ± 0.7	5.6 ± 0.5	-16.0 ± 0.3	4.8 ± 0.2	-14.1 ± 0.2			5.9 ± 0.3	-15.3 ± 0.6		5.7 ± 0.1		-15.4 ± 0.4		7.3 ± 0.2			-16.3 ± 0.6		7.2 ± 0.2	-15.3 ±0.8	5.9 ± 0.9
Δ^host-photosymbionts^	0.20 ± 0.046	3.37 ± 0.78	0.60 ± 0.10	2.33 ± 0.39	0.45 ± 0.07			2.06 ± 0.40	-1.32 ± 0.43		2.46 ± 0.34		0.19 ± 0.05		2.02 ± 0.10			0.36 ± 0.09		2.56 ± 0.52	0.11 ±0.66	2.51 ± 0.64
***P. meandrina***																						
Polyp and photosymbiont	-12.9 ± 0.7	5.5 ± 0.2	-13.3 ± 1.0	5.1 ± 0. 3	n.d.			n.d.	n.d.		n.d.		-14.1 ± 0.1		6.9 ± 0.3			-15.4 ± 0.8		6.5 ± 0.4	-13.8 ± 1.6	5.9 ± 0.8
Photosymbiont	-13.5 ± 0.7	3.4 ± 0.8	-13.9 ± 0.6	3.5 ± 0.4	n.d.			n.d.	n.d.		n.d.		-14.5 ± 0.2		5.8 ± 0 .1			-15.5 ± 0.6		5.8 ± 0.0	-14.2 ± 0.9	4.5 ± 1.3
Polyp	-13.2 ± 0.6	6.2 ± 0.2	-13.6 ± 0.6	5.8 ± 0.4	n.d.			n.d.	n.d.		n.d.		-14.0 ± 0.1		6.9 ± 0.1			-15.9 ± 0.6		6.9 ± 0.2	-14.1 ± 1.1	6.4 ± 0.5
Δ^host-photosymbionts^	0.32 ± 0.09	2.76 ± 0.78	0.25 ± 0.06	2.31 ± 0.55	n.d.			n.d.	n.d.		n.d.		0.48 ± 0.15		1.07 ± 0.08			-0.37 ± 0.09		1.02 ± 0.16	0.19 ± 0.32	1.90 ± 0.91
***P. verrucosa***																						
Polyp and photosymbiont	-13.5 ± 0.7	4.4 ± 1.0	-13.3 ± 0.5	5.0 ± 0.3	-12.9 ± 0.5			5.1 ± 0.1	-14.3 ± 0.6		5.0 ± 0.6		-15.0 ± 0.7		6.6 ± 0.5			-15.4 ± 0.6		6.5 ± 0.1	-14.1 ± 1.1	5.5 ± 0.9
Photosymbiont	-12.9 ± 0.6	3.9 ± 0.1	-12.7 ± 0.3	3.2 ± 0.4	-13.8 ± 0.5			3.6 ± 0.1	-14.3 ± 0.9		4.0 ± 0.6		-15.5 ± 0.5		4.9 ± 0.2			-15.3 ± 0.5		5.0 ± 0.7	-14.2 ± 1.2	4.2 ± 0.7
Polyp	-13.3 ± 0.7	5.9 ± 0.3	-13.5 ± 0.5	5.2 ± 0.3	-13.4 ± 0.5			5.5 ± 0.3	-14.5 ± 0.9		5.4 ± 0.5		-15.4 ± 0.5		6.9 ± 0.4			-15.9 ± 0.4		7.2 ± 0.5	-14.4 ± 1.2	6.1 ± 0.9
Δ^host-photosymbionts^	-0.42 ± 0.15	1.96 ± 0.32	-0.86 ± 0.20	1.97 ± 0.39	0.47 ± 0.06			1.84 ± 0.19	-0.19 ± 0.07		1.38 ± 0.37		0.07 ± 0.07		2.01 ±0.33			-0.53 ± 0.08		2.20 ± 0.59	-0.21 ± 0.44	1.89 ± 0.43
***P. cactus***																						
Polyp and photosymbiont	-14.9 ± 0.7	4.2 ± 0.5	-14.5 ± 0.3	4.3 ± 0.2	-14.1 ± 0.7			4.5 ± 0.7	-13.8 ± 0.6		4.5 ± 0.1		-17.3 ± 0.3		5.9 ± 0.3			-17.1 ± 0.4		6.1 ± 0.2	-15.4 ± 1.5	5.1 ± 0.8
Photosymbiont	-15.3 ± 0.3	3.1 ± 0.4	-15.0 ± 0.5	3.6 ± 0.3	-14.3 ± 0.4			3.5 ± 0.3	-16.0 ± 0.6		4.4 ± 0.4		-17.8 ± 0.2		5.0 ± 0.2			-16.9 ± 0.4		5.6 ± 0.1	-15.9 ± 1.3	4.2 ± 0.9
Polyp	-14.9 ± 0.2	4.5 ± 0.2	-14.4 ± 0.4	4.4 ± 0.3	-13.9 ± 0.4			4.7 ± 0.3	-15.1 ± 0.8		5.1 ± 0.4		-17.5 ± 0.2		5.8 ± 0.2			-17.1 ± 0.4		6.6 ± 0.3	-15.6 ± 1.4	5.1 ± 0.5
Δ^host-photosymbionts^	0.40 ± 0.04	1.13 ± 0.34	0.62 ± 0.13	0.87 ± 0.19	0.33 ± 0.06			1.18 ± 0.20	0.94 ± 0.27		0.73 ± 0.11		0.31 ± 0.08		0.79 ± 0.23			-0.24 ± 0.03		0.96 ± 0.21	0.37 ± 0.38	1.00 ± 0.32
***M. tuberculosa***																						
Polyp and photosymbiont	-14.1 ± 0.4	4.0 ± 0.5	-13.1 ± 0.6	4.2 ± 0.1	-14.2 ± 1.0			4.6 ± 0.3	-13.5 ± 0.1		4.4 ± 0.1		-16.0 ± 0.6		5.5 ± 0.5			-15.2 ± 0.4		5.9 ± 0.6	-14.3 ± 1.1	4.7 ± 0.8
Photosymbiont	-15.2 ± 0.4	3.4 ± 0.2	-13.8 ± 0.6	3.2 ± 0.4	-15.2 ± 0.6			3.5 ± 0.4	-11.5 ± 0.3		3.5 ± 0.1		-15.1 ± 0.2		5.4 ± 0.3			-15.1 ± 0.4		5.3 ± 0.6	-14.4 ± 1.4	3.9 ± 0.9
Polyp	-13.8 ± 0.5	4.8 ± 0.3	-12.8 ± 0.6	4.5 ± 0.6	-13.7 ± 0.6			5.3 ± 0.0	-12.9 ± 0.3		5.0 ± 0.4		-14.4 ± 0.3		6.5 ± 0.2			-16.1 ± 0.1		7.3 ± 0.7	-13.9 ± 1.2	5.5 ± 1.1
Δ^host-photosymbionts^	1.41 ± 0.33	1.36 ± 0.35	0.97 ± 0.22	1.27 ± 0.48	1.48 ± 0.31			1.80 ± 0.36	-1.44 ± 0.23		1.51 ± 0.43		0.71 ± 0.14		1.09 ± 0.12			-0.99 ± 0.28		1.95 ± 0.13	0.11 ± 1.17	1.51 ± 0.42
**Mean**																						
Polyp and photosymbiont	-13.3 ± 1.6	4.9 ± 0.6	-13.5 ± 1.5	4.7 ± 0.5	-13.4 ± 0.9			5.0 ± 0.5	-13.0 ± 1.5		4.7 ± 0.5		-14.7 ± 1.7		6.6 ± 0.7			-15.1 ± 1.6		6.3 ± 0.6	-13.8 ±1.7	5.3 ± 0.9
Photosymbiont	-13.7 ± 1.6	3.8 ± 1.1	-13.8 ± 1.6	3.4 ± 0.8	-14.1 ± 0.6			4.2 ± 0.9	-13.0 ± 1.8		3.9 ± 0.8		-15.2 ± 1.3		5.8 ± 1.3			-15.0 ± 1.6		5.7 ± 0.8	-14.1 ± 1.6	4.4 ± 1.3
Polyp	-13.2 ± 1.6	5.3 ± 0.6	-13.5 ± 1.3	5.0 ± 0.6	-13.6 ± 0.7			5.5 ± 0.5	-13.4 ± 1.5		5.5 ± 0.4		-14.3 ± 1.7		6.7 ± 0.7			-15.3 ± 1.6		7.0 ± 0.6	-13.8 ± 1.6	5.8 ± 0.9
Δ^host-photosymbionts^	0.53 ± 0.56	1.57 ± 1.12	0.24 ± 0.56	1.56 ± 0.84	0.53 ± 0.63			1.32 ± 0.65	-0.35 ± 0.75		1.56 ± 0.73		0.84 ± 0.87		1.27 ± 1.10			-0.30 ± 0.47		1.27 ± 0.79	0.27 ± 0.76	1.40 ± 0.90

n.d. means not detected when coral species was absent.

Stable isotopic composition of coral host tissues showed spatial variations (Table **4**). Coral host tissues were most ^13^C-depleted and ^15^N-enriched at Vaiare compared with Tiahura and Maharepa during both sampling series (Table **6** and Figure **5**). Temporal variations of coral δ^13^C values differed according to the site considered (Table **4**), but the low amplitude of variations (less than 1‰) was not biologically meaningful. No temporal effect was observed for δ^15^N values of coral host tissues at the three sites (Table **4**). For each coral species, spatial and temporal variations (and interactions between factors site and season) were tested independently (Table **7**). Results indicated that carbon and nitrogen isotopic composition of coral host tissues varied among sites and between collection times, but these variations were species-specific.

**Table 7:** Significance of spatial and temporal variations of stable isotope values (δ^13^C and δ^15^N) of coral host tissues and stable isotope differences between coral host tissues and their associated photosymbionts (Δ^host-photosymbionts 13^C and Δ^host-photosymbionts 15^N) tested with PermANOVA for each species.

Species	*P. rus*		*N. irregularis*		*A. cytherea*		*A. hyacinthus*		*A. pulchra*		*P. damicornis*		*P. meandrina*		*P. verrucosa*		*P. cactus*		*M. tuberculosa*	
	F	*p*	F	*p*	F	*p*	F	*p*	F	*p*	F	*p*	F	*p*	F	*p*	F	*p*	F	*p*
	(df)		(df)		(df)		(df)		(df)		(df)		(df)		(df)		(df)		(df)	
δ^13^C																				
Site	28.4	***	5.6	*	7.6	*	15.2	**	48.6	***	9.0	**	28.1	**	27.8	***	19.1	***	37.8	***
	(2)		(1)		(1)		(1)		(2)		(2)		(1)		(2)		(2)		(2)	
Season	6.9	*	0.3	n.s.	50.4	**	2.6	n.s.	22.9	**	22.0	**	13.4	**	5.9	*	0.0	n.s.	0.2	n.s.
	(1)		(1)		(1)		(1)		(1)		(1)		(1)		(1)		(1)		(1)	
Site*Season	1.4	n.s.	0.1	n.s.	1.2	n.s.	6.3	n.s	49.3	**	0.2	n.s.	5.7	*.	17.1	***	1.4	**	17.5	**
	(2)		(1)		(1)		(1)		(2)		(2)		(1)		(2)		(2)		(2)	
Residuals	24		14		8		8		16		14		10		16		16		14	
δ^15^N																				
Site	67.5	***	4.5	n.s.	39.9	**	20.4	**	29.2	***	85.3	***	38.7	***	39.5	***	73.8	***	49.1	***
	(2)		(1)		(1)		(1)		(2)		(2)		(1)		(2)		(2)		(2)	
Season	0.2	n.s.	4.5	n.s.	1.2	n.s.	1.6	n.s.	2.4	n.s	8.3	*	3.0	n.s.	0.5	n.s.	10.0	**	0.0	n.s
	(1)		(1)		(1)		(1)		(1)		(1)		(1)		(1)		(1)		(1)	
Site*Season	0.2	n.s.	2.2	n.s.	1.8	n.s.	4.4	n.s.	1.0	n.s	2.8	n.s.	1.5	n.s.	2.3	n.s.	4.2	*	3.1	n.s
	(2)		(1)		(1)		(1)		(2)		(2)		(1)		(2)		(2)		(2)	
Residuals	24		14		8		8		16		14		10		16		16		14	
Δ^host-photosymbionts 13^C																				
Site	5.6	*	9.6	**	5.1	n.s.	12.8	**	44.0	***	41.7	***	18.8	**	92.3	***	49.7	***	54.5	***
	(2)		(1)		(1)		(1)		(2)		(2)		(1)		(2)		(2)		(2)	
Season	40.8	***	6.6	**	37.9	***	34.1	**	224.4	***	16.2	**	59.2	***.	164.1	***	0.8	n.s.	176.2	***
	(1)		(1)		(1)		(1)		(1)		(1)		(1)		(1)		(1)		(1)	
Site*Season	12.4	***	1.6	n.s.	39.7	***	4.4	n.s.	38.0	***	73.0	***	51.1	***	1.9	n.s.	42.6	***	38.2	***
	(2)		(1)		(1)		(1)		(2)		(2)		(1)		(2)		(2)		(2)	
Residuals	24		14		8		8		16		14		10		16		16		14	
Δ^host-photosymbionts 15^N																				
Site	0.4	n.s.	14.5	**	13.1	*	4.0	n.s.	11.0	***	3.2	n.s.	27.6	**	3.4	n.s.	3.8	*	1.6	n.s.
	(2)		(1)		(1)		(1)		(2)		(2)		(1)		(2)		(2)		(2)	
Season	22.6	***	9.4	**	75.9	***	31.3	**	0.5	*	0.4	n.s.	0.9	n.s.	0.3	n.s.	6.2	*	0.7	n.s.
	(1)		(1)		(1)		(1)		(1)		(1)		(1)		(1)		(1)		(1)	
Site*Season	6.0	**	9.1	*	6.7	*	55.6	***	47.5	**	5.6	*	0.5	n.s.	1.4	n.s.	7.2	**	4.6	*
	(2)		(1)		(1)		(1)		(2)		(2)		(1)		(2)		(2)		(2)	
Residuals	24		14		8		8		16		14		10		16		16		14	

* means *p* ≤ 0.5, ** *p* ≤ 0.01, *** *p* ≤ 0.001 and n.s. not significant.

#### Relationship between coral hosts and their associated Symbiodinium

δ^13^C and δ^15^N values of photosymbionts were correlated with those of the coral host tissues, clearly indicating trophic relationships (δ^13^C_photosymbionts_ = -1.48 + 0.91δ^13^C_coral host_, r = 0.88, N = 192, *p* ≤ 0.001 and δ^15^N_photosymbionts_ = -1.46 + 1.01δ^15^N_coral host_, r = 0.73, N = 192, *p* ≤ 0.001; graphics not shown). Stable isotope shifts between each coral host and its associated photosymbionts (Δ^host-photosymbionts 13^C and Δ^host-photosymbionts 15^N) were determined to further explore the translocation of fixed carbon and nitrogen from photosymbionts to coral hosts. Despite the good correlation between stable isotope values of photosymbionts and coral host tissues, Δ^host-photosymbionts 13^C and Δ^host-photosymbionts 15^N showed significant differences among coral species (Table **4**). Δ^host-photosymbionts 13^C ranged from -1.44 ± 0.23‰ (*M. tuberculosa* at Maharepa during the dry season) to 2.98 ± 0.58‰ (*A. pulchra* at Vaiare during the wet season), and Δ^host-photosymbionts 15^N ranged from 0.10 ± 0.03‰ (*A. cytherea* at Tiahura during the wet season) to 3.90 ± 0.28‰ (*A. pulchra* at Vaiare during the wet season, Table **6**). Considering all sites and both seasons, Δ^host-photosymbionts 13^C in *P. rus* and *P. verrucosa* were significantly lower than those of *A. cytherea*, *M. tuberculosa* and *N. irregularis* (post-hoc multiple comparison test, *p* ≤ 0.05). *P. rus, A. hyacinthus* and *A. cytherea* had smaller Δ^host-photosymbionts 15^N compared to *N. irregularis*, *P. damicornis*, *P. meandrina*, *P. verrucosa*, *A. pulchra* and *M. tuberculosa* (post-hoc multiple comparison test, *p* ≤ 0.05; Table **6**). 

Spatial and temporal variations of Δ^host-photosymbionts 13^C were statistically significant (Table **4**), but Δ^host-photosymbionts 13^C remained low (0.27 ± 0.76‰) at all sites and during both collection times. Δ^host-photosymbionts 15^ N did not show any spatial or temporal variations (mean Δ^15^N = 1.4 ± 0.90‰). The influences of spatial and temporal variations (and interactions between factors site and season) on Δ^host-photosymbionts 13^C and Δ^host-photosymbionts 15^N were tested independently for each coral species (Table **7**). No consistent patterns were observed and spatial and temporal changes were species-specific.

## Discussion

### Spatial variations in stable isotopic composition of corals related to their associated photosymbionts

Our results have highlighted that corals from Vaiare, a turbid sedimentary and phytoplankton-rich site, were most ^13^C-depleted and ^15^N-enriched relative to the corals from the two other sites, Tiahura and Maharepa, during both collection times. Such differences in δ^13^C and δ^15^N values of both coral host tissues and photosymbiont might be explained by changes in (1) the degree of coral heterotrophy, (2) stable isotope values of the sources of carbon and nitrogen assimilated by photosymbionts, and/or (3) the mechanisms by which the sources were fractionated.

#### Degree of coral heterotrophy

Carbon and nitrogen isotopic compositions of corals reflect the assimilation of different sources of nutrition including photosymbiont-derived carbon and nitrogen and heterotrophic prey. Experimental and *in situ* studies have shown that if coral hosts incorporate carbon from sources other than photosymbionts, δ^13^C values of both coral host tissues and photosymbiont approach those of SPOM and differences in δ^13^C values between coral host tissues and their associated photosymbionts (Δ^host-photosymbionts 13^C ) increase [8,20,41]. ^13^C-depletion at Vaiare could have resulted from corals deriving more of their carbon through heterotrophy, as δ^13^C values of corals (mean δ^13^C = -14.9 ± 1.6‰) tend to follow those of very ^13^C-depleted SPOM at around -21.2‰. Vaiare is the ferry area of Moorea Island, where higher phytoplankton concentrations (i.e. Chl *a*, Figure **2A**) were measured in the seawater column due to additional nutrients in this area from sediment resuspension (i.e. SPIM enrichment, Figure **2B**). Heterotrophy by corals can be enhanced by the increase of available particulate food in their turbid environments, to counteract the reduction in phototrophy by the photosymbionts and allow the corals to maintain a positive energy budget [42]. However, mean Δ^host-photosymbionts 13^C at Vaiare remained small (0.27‰) and was similar to that of Tiahura (0.39‰), indicating that if all corals together are considered, either there was no increase of heterotrophy, or carbon isotope evidence for increased heterotrophy was masked by a rapid recycling of carbon between host and photosymbionts, as suggested by Einbinder et al. [43]. The lack of increased heterotrophy by corals living at the turbid and nutrient-rich site of Vaiare was also confirmed by δ^15^N values. Ingestion of SPOM may represent an important source of nitrogen for corals living in shallow inshore waters [3], and when the contribution of heterotrophy increases, δ^15^N of corals approaches those of SPOM [21,44]. However, in our study of Moorea Island, corals were ^15^N-enriched at Vaiare relative to the corals from Tiahura and Maharepa, and Δ^host-photosymbionts 15^N revealed low variability among sites and averaged +1.4‰, thus suggesting that the degree of heterotrophy at Vaiare was not enhanced. Similar ranges of ^15^N-enrichment with the change in trophic level between coral host tissues and photosymbionts have been reported by Swart et al. [22], supporting the hypothesis of the recycling of internal ammonia and amino acids between the host and photosymbiont suggested by Reynaud et al. [44]. Our results confirmed that increased heterotrophy by coral hosts in turbid rich nutrient areas is not a universal pattern. Stable isotopic compositions of some species showed variability through space and time, suggesting that adjustments in the heterotrophic pathway is a species-specific phenomenon [7,30,45,46].

#### Sources of carbon and nitrogen assimilated by photosymbionts and mechanisms of fractionation

Rather than the degree of heterotrophy, ^13^C-depletion and ^15^N-enrichment of corals from Vaiare relative to the corals from the two other sites may be better explained by the isotopic values of the dissolved carbon and nitrogen sources assimilated by photosymbionts and the mechanisms by which the sources are fractionated related to the degree of light available in such a sedimentary and turbid environment [23,25,47,48]. Indeed, algae living in symbiosis with corals use two principal sources of carbon for photosynthesis: CO_2_ from animal metabolism and the external pool of bicarbonate (HCO^3-^) [8]. In our study, the δ^13^C value of CO_2_ originating from the coral hosts was about -13.9‰ (represented by the mean δ^13^C of coral hosts; Table **6**). Using the equation from Rau et al. [49], δ^13^C of CO_2_ resulting from equilibrium fractionation of HCO_3_^-^ from external seawater was about -7‰. Several studies have shown that δ^13^C values of corals under high levels of light are relatively positive, and become more negative as light intensity decreases [8,9,30]. Under high levels of light, photosynthetic rates are high and all available CO_2_ is fixed by photosymbionts, inducing the reduction of carbon isotopic discrimination. Thus, the δ^13^C values of the photosymbionts approach those of their carbon sources (i.e. the coral hosts) [50]. Moreover, CO_2_ from animal metabolism is totally consumed, and photosymbionts must use larger fractions of CO_2_ from the internal tissular bicarbonate pool [8]. The combination of the reduction of carbon isotopic discrimination and the increased proportion of CO_2_ utilized from the bicarbonate pool induce a relative enrichment of photosymbiont δ^13^C under high light levels. Moreover, a similar ^13^C-enrichment is observed in coral host tissues due to the translocation of fixed carbon from the photosymbionts. Our findings support the hypothesis that corals living in sedimentary and turbid environment with reduced light levels at Vaiare were generally more ^13^C-depleted compared to corals from clear environment at Tiahura and Maharepa.

The isotopic composition of dissolved inorganic carbon (DIC) also contributes to inter-reef variability in coral δ^13^C values [23], and δ^13^C values of DIC are generally correlated with the occurrence of primary production which removes isotopically light carbon from the seawater [51]. At Moorea Island, Chl *a* concentrations in the seawater were highest at Vaiare during both sampling times (Figure **2A**) and negatively correlated with δ^13^C values of SPOM (Figure **4A**). The remineralization of detritus by benthic bacteria at the surface of the sediment, and the subsequent resuspension of this detritus with the circulation of ferry boats past Vaiare, have caused further depletion in δ^13^C of the DIC pool in this area. Lighter carbon was thus likely fixed and translocated by the primary producers at Vaiare to higher trophic levels (i.e. corals).

Our results also revealed that all coral species considered were significantly ^15^N-enriched at the turbid site of Vaiare compared to Tiahura and Maharepa. We would have expected a ^15^N-depletion at this turbid site, since Muscatine and Kaplan [21] found a positive relationship between depth (i.e. low light and particulate nutrient enrichment) and ^15^N-depletion. Indeed, low light exposure decelerates photosynthetic rates, which in turn decreases the internal demand for nitrogen and increases fractionation [21,36,52]. Predation on constantly depleted zooplankton also contributes to the ^15^N-depletion in coral tissue [41]. The observed ^15^N-enrichment of coral tissues from Vaiare thus doesn’t suggest a light and/or feeding effect but rather supports different isotopic composition of DIN sources between sites [23,52]. δ^15^N values of DIN should be affected by total primary production on the reef since autotrophic organisms discriminate against ^15^NO^3-^ [53,54], but our results showed that δ^15^N values of SPOM were not correlated with Chl *a* concentrations (Figure **4B**). δ^15^N values of DIN are generally higher (by up to 5‰) at eutrophic sites, with a concurrent transfer of this enrichment being apparent in primary producers and higher trophic levels [24,55,56]. Enriched δ^15^N values of marine organisms are not necessarily the reflection of sewage or ground water impacts [22], and at Vaiare waste water discharges were negligible as confirmed by NO^3-^ concentrations. Other biotic processes in marine ecosystems can lead to large variations in the stable isotopic composition of the DIN pool (see Peterson and Fry [15] for review). In particular, denitrification processes induce the loss of isotopically light ^14^N from the DIN pool, causing the remaining nitrate pool to be ^15^N-enriched [57]. Sediment resuspension affects this process, as Sloth et al. [58] showed that denitrification rates were stimulated in resuspended mesocosms relative to controls. SPOM and coral ^15^N-enrichment at Vaiare were likely due to increased bacterial denitrification processes leading to ^15^N-enrichment of DIN; these effects probably dominated and masked other potential influencing factors on nitrogen stable isotope ratios in corals.

### Temporal variations in the stable isotopic composition of corals

The δ^13^C values of coral host tissues and Δ^host-photosymbionts 13^C showed significant temporal variations (Tables **4** and **6**). However, the differences were small (less than 1‰) and so may not be biologically meaningful. Moreover, no temporal effect was observed in δ^15^N values of coral host tissues and Δ^host-photosymbionts 15^N. These results indicated that heterotrophy was not enhanced during the cloudy wet season when the densities of photosymbionts in coral tissues were lower and the concentration of phytoplankton in the surrounding seawater was higher. Corals from Moorea Island relied principally on photosynthates translocated by their photosymbionts during both sampling times. Few researchers have investigated seasonal changes in the nutrition of corals using stable isotope ratios. Swart et al. [28] suggested that δ^13^C values in coral host tissues, which were collected during summer months were isotopically, more positive than those measured at the end of the summer. The causes of intra annual variations in tδ^13^C are believed to be related to carbon limitation and decreased fractionation of the inorganic carbon pool during the early summer months. Statistically significant seasonal variations in the δ^13^C of coral host tissues and photosymbionts from the coral species *Montastraea faveolata* were confirmed by Swart et al. [22], at one of the site investigated in the Florida Keys. The authors [22] assumed that the absence of similar signals at all the sites investigated may, in part, be a result of the use of different individuals and therefore may represent interspecimen variability due to slightly different inherent physiology of the different coral colonies studied. However, in our study of Moorea Island, coral fragments were collected from the same colonies during both wet and dry seasons to prevent potential sampling artifacts. Swart et al. [22] also reported that Δ^host-photosymbionts 13^C became minimized when photosymbiont densities were at their lowest. In their study SPOM did not contribute substantially to the budget of *M. faveolata* as δ^15^N values of the coral host tissues were very depleted compared to the SPOM values. The lack of clear temporal changes in the stable isotope values of the scleractinian corals from Moorea Island and the low Δ^host-photosymbionts 13^C values (about 0‰) were probably due to small changes in environmental parameters during 2011. Annual variations of environmental parameters (light and nutrients) in New Caledonia lagoon were described as weak compared to short term variations [59]. A long-time survey to follow such temporal variations would confirm our preliminary observations.

### Inter-specific variations in acclimation of corals to their environments

Data collected on the most abundant species of corals living in shallow fringing reefs around Moorea Island showed similar ranges of δ^13^C and δ^15^N to those previously reported for other tropical scleractinian corals [8,21]. Spatial differences in stable isotope ratios of corals resulted from changes in the sources of carbon and nitrogen assimilated by photosymbionts and the influence of light on source fractionation. Clear temporal variations of coral stable isotope values were not observed at Moorea Island. However, when considering each coral species separately, their isotopic compositions did not show the same variability among sites and between collection times (i.e. results were species-specific). Differences in carbon and nitrogen isotopic ratios among coral species reflect the multitrophic pathways used by corals, and/or their different physiological adaptations involving photosynthesis, respiration and assimilation rates of dissolved inorganic nutrients [8,9,13,21,30]. It is clear that SPOM capture and feeding rates by corals vary among species in relation to their surface area [60]. However, Δ^host-photosymbionts 13^C values recorded in coral species from Moorea Island were low (from -1.44 ± 0.23‰ to 2.98 ± 0.58‰) compared to those for heterotrophic corals (-8‰) living in deep environments [8]. Muscatine et al. [8] attributed interspecific differences in δ^13^C values to varying resistance of coral tissues to diffusion of CO_2_ and HCO^3-^. Reduced diffusion distances increase the replenishment of internal DIC and favor a stronger isotopic discrimination. For example, depleted δ^13^C values in *Madracis auretenra* (-16‰) were due to thick coral tissues and low production of mucus reducing diffusion distances [30]. Internal CO_2_ depletion could also be exacerbated by the high cell densities of photosymbionts [61]. At Moorea Island, corals from the genera *Pocillopora*, *Napopora*, *Pavona* and *Montipora* were ^13^C-depleted compared to corals from the genera *Porites* and *Acropora*, but our results did not indicate any relationship between the density of photosymbionts and coral δ^13^C values (data not shown). δ^13^C values of *P. rus*, *A. cytherea*, *A. hyacinthus* and *P. damicornis* remained relatively similar through space and time (Table **6**), indicating that these coral species did not adjust their physiology to changing environmental conditions. Conversely, stable isotopic compositions of *P. cactus*, *M. tuberculosa*, *P. meandrina* and *P. verrucosa* showed large variations among sites and/or between times, thus indicating a physiological plasticity of these species. To further improve our understanding of the effects of space and time on the isotope compositions of different coral species and their associated photosymbionts, complementary data on coral physiology would be of great interest.
